# Physical activity and combined hormonal contraception: association with female students’ perception of menstrual symptoms

**DOI:** 10.3389/fphys.2023.1185343

**Published:** 2023-05-17

**Authors:** Valérie Bougault, Sandrine Schiano-Lomoriello, Carole Castanier, Corinne Buisson, Magnus Ericsson, Caroline Teulier, Katia Collomp

**Affiliations:** ^1^ LAMHESS, Université Côte d’Azur, Nice, France; ^2^ CIAMS, Université d'Orléans, Orléans, France; ^3^ CIAMS, Université Paris-Saclay, Orsay, France; ^4^ Sport, Physical Activity, Rehabilitation and Movement for Performance and Health Research Group, Orléans, France; ^5^ Laboratoire Anti-Dopage Français, LADF, Université Paris-Saclay, Chatenay-Malabry, France

**Keywords:** exercise, female, menses, contraception, dysmenorrhea, premenstrual syndrome

## Abstract

**Aim:** The aim of this study was to examine the association between physical activity (PA) and combined hormonal contraceptive (CHC) on female students’ self perceptio of their menstrual cycle symptoms.

**Methods:** Healthy French female students (*n* = 834) completed an online questionnaire to assess their PA level (Group 1: non-active; Group 2: moderate physical activity; Group 3: high physical activity; Group 4: very high physical activity), menstrual status or contraception use, self-reported diet and medication, impact on engagement in some social activities, and self-assessment of perceived mental and physical symptoms during the week prior to menses (PM) for students with a normal menstrual cycle (NMC), and the week of menses (ME) for normal menstrual cycle students and those using combined hormonal contraception.

**Results**: Whatever the conditions (PM and ME, NMC and CHC), fewer self-perceived symptoms and self-reported alteration in fat intake were reported by the students in Group 4, and more analgesic and anti-inflammatory medication use was reported by Group 1. Fewer self-perceived symptoms were also found in CHC vs NMC female students for all physical activity levels, but in a more marked way when associated with very high physical activity. In addition, less university and sports practice absenteeism was observed with high and very high physical activity.

**Conclusion:** In conclusion, the perception of menstrual cycle symptoms was lower with very high physical activity, as with combined hormonal contraception. Moreover, female students training more than 5 h/week also reported less university absenteeism and impairment in physical activities. Further studies are necessary to establish the causal link of physical activity and combined hormonal contraception on menstrual symptoms.

## Introduction

The menstrual cycle may significantly affect females’ health, daily activities, quality of life and performance (Tadakawa et al., 2016; Yu et al., 2017; McNulty et al., 2020; Castanier et al., 2021; Mitsuhashi et al., 2022; Pogodina et al., 2022). Symptoms are not limited to females with menstrual disorders, such as oligomenorrhea and amenorrhea, but are also reported by females with a normal menstrual cycle (NMC) (Roomruangwong et al., 2021; Righi and Barroso, 2022). During the late luteal phase, i.e., the premenstrual phase (PM), a systematic review reported a pooled prevalence of 51.3% of premenstrual syndrome in female students, with various reported self-perceived symptoms such as cyclic mood disorders, somatic or affective disorders, and severe fatigue (Maity et al., 2022). This prevalence varies from 32% to 95%, with a majority of studies conducted outside Europe (Yamamoto et al., 2009; Pinar et al., 2011; Hamaideh et al., 2014; Tolossa and Bekele, 2014; Romito et al., 2017; Bhuvaneswari et al., 2019; Hashim et al., 2019; Maity et al., 2022). Always among female students, 72.7% report dysmenorrhea, i.e., painful menstrual cramps and other associated symptoms (low back pain, headache, vomiting, diarrhea, fatigue, or nausea) during the early follicular phase, occurring the first days of menses (ME) (De Sanctis et al., 2016). This prevalence varies from 24% to 98% (Maity et al., 2022), and in Europe, a recent study led in Spain reported a prevalence of 76% among university student (Fernández-Martínez et al., 2019a). Thirteen to 70% of females take hormonal contraception (HC), with a large majority taking oral combined estrogen-progestin oral contraceptives (CHC) (Martin et al., 2018; Oxfeldt et al., 2020). In general, hormonal contraception pills are taken every day for 21 days, followed by a 7-day washout to reproduce a theoretical cycle of 28 days, but with more stable hormone levels. CHC users also report fewer self-perceived symptoms than females with a NMC (Sadler et al., 2010; Brown et al., 2017). Indeed, various hormonal oral contraception, may be used as the treatment of dysmenorrhea, despite limited evidence on pain improvement (Wong et al., 2009), or controversial results on menstrual bleeding, physical and mood symptoms (Sadler et al., 2010; Bahamondes et al., 2015).

According to a recent study, 68% of oral contraception users reported taking it for a contraceptive purpose and 32% for a therapeutic purpose (Nielson and Beltz, 2021). However, very little literature exists on this point so it cannot be excluded that females use HC for both reasons. It is not uncommon for females to manipulate oral contraception by intentionally skipping the 7-day washout to avoid having menses and associated menstrual pain (Schaumberg et al., 2018). The use of non-drug therapies, including regular physical activity, is also reported to limit symptoms during both the PM and ME phases, both in combination with HC and not (Matthewman et al., 2018; Armour et al., 2019a; Armour et al., 2019b; Maity et al., 2022; Ravichandran and Janakiraman, 2022). However, there is currently insufficient information to propose a consensus regarding the best physical activity type, frequency, intensity or program to reduce the frequency and severity of symptoms during both phases (Matthewman et al., 2018; Armour et al., 2019a; Armour et al., 2019b; Ravichandran and Janakiraman, 2022). The combination of two systematic reviews on randomized control trials suggested that whatever the intensity, 30–60 min of aerobic exercise three to five times a week for about 10 weeks may be effective in reducing physical and psychological symptoms during the PM and ME phases (Armour et al., 2019a; Ravichandran and Janakiraman, 2022). However, only few studies were selected and only effects of light intensity (stretching or yoga postures) or aerobic exercise interventions were reported in these meta-analyses. A lack of a positive effect of physical exercise or intense sports activity has also been observed by some authors, as has an aggravation of self-perceived symptoms during the PM phase in some females not taking HC (Czajkowska et al., 2015; Kroll-Desrosiers et al., 2017; Witkoś and Hartman-Petrycka, 2021). Also, very few studies have been done on the potential benefits of physical activity on mental and physical symptoms during the ME phase, with some studies reporting and others not reporting a decrease in dysmenorrhea (Kishali et al., 2006; Armour et al., 2019a; Huang et al., 2022) in athletic and non-athletic females not using HC. To our knowledge, no study has investigated the synergistic impact of physical activity and CHC.

Finally, dietary changes have been reported by 89% of female students, in favor of craving sweets during PM, with high calories/fat/sugar/salt foods being associated with physical symptoms (Hashim et al., 2019). Exercise training appears to be accompanied by positive changes in food preferences in line with an overall improvement in appetite control (Beaulieu et al., 2020), and we think it may reduce sugar/fat cravings during PM, although currently controversial (Maged et al., 2018; Mohebbi Dehnavi et al., 2018). To relieve their symptoms, either during PM or ME, painkillers, such as non-steroidal anti-inflammatory drugs (NSAIDS) and hot drinks are amongst the most common measures taken by the female students, and 61% have been reported to use analgesic drugs (Maity et al., 2022). The absence of physical activity being often associated to dysmenorrhea (Maity et al., 2022), it is possible that physical activity reduces cortisol and prostaglandins, and the need for NSAIDS. It is to note that no study compared the effect of the level of physical activity on medication and self-reported diet changes during PM and ME.

Therefore, the aim of this study, based on an online questionnaire, was to examine self-perceived mental and physical symptoms, self-reported diet and medication in NMC female students during both the PM and ME phases regarding physical activity. At the same time, we sought to assess the potential synergistic effects of CHC use during the ME phase. We hypothesized that physical activity alone or combined to CHC is associated with decrease in female students’ perception of menstrual symptoms.

## Methods

This research was approved by the Paris-Saclay Ethics of Research Committee (CER-Paris-Saclay-2021-098). The study was carried out by means of an online questionnaire in French.

### Participants

From December 2021 to March 2022, female French students were invited to complete an anonymous online questionnaire about their menstrual cycle and CHC use. Participants were recruited through student services of the Universities of Orleans and Paris-Saclay via mailing lists and on social media. The inclusion criteria were as follows: 1) female student between 18 and 28 years of age; 2) healthy with no chronic therapeutic treatment except for menstrual symptoms; and 3) consistent physical activity and contraception over the past year.

### Questionnaire

The questionnaire was exclusively in French and designed to take about 15 min to complete.

The questionnaire was made up of six sections with most closed-ended questions:

1) **General information:** After given consent, all participants reported their age, height, body mass, age of menarche, main discipline of their university/college studies, HC use (with reasons) or not, and had to select their physical activity level: <1 h/week; 1–5 h/week; 5–10 h/week; >10 h/week;
2) **Self-perceived symptoms:** Several closed-ended questions about their physical and mental self-perceived symptoms during the PM (NMC) and ME (NMC and CHC) were then proposed from a list of symptoms; To assist comprehension, the menstrual cycle was split into weeks: last week of the cycle in NMC for PM phase (PM_NMC_); week of menses (start of bleeding) for ME phase, i.e., first week of the cycle in NMC (ME_NMC_) or week of washout (ME_CHC_). As both PM and ME phases occurred the same week in oral CHC users, to make it easier to fill out the questionnaire, we focused on the symptoms starting with menses (ME_CHC_). The females answered “yes” or “no” for symptoms. When they answered “yes,” they were asked to specify their physical and/or mental symptoms from a list of symptoms. In case they wanted to report a new symptom, they could check the “other” box and describe the symptom.
3) **Self-reported diet, complement and medication:** Dietary, complement and medication behaviors during these phases, i.e., questions on more/less total food intake, more/less sweet and fat intake, more/less supplements and medication (NSAIDS and painkillers) were asked;
4) **School and physical activity:** The perceived impact of the PM and/or ME phases on university attendance and physical activity practice were assessed, specifying which ones were impacted and why (open-ended questions);
5) **Interaction with Physical activity:** Participants were asked whether they have increased/decreased/no change in symptoms when practicing PA during PM and ME;
6) **Feminine hygiene product use:** Participants were asked to give their choice of feminine hygiene products (menstrual pads, cups, underwears or tampons) at rest and during the physical activity.

We divided the responses into the following groups based on the participants’ physical and sports activity (Table 1). Group 1: non-active (<1 h/week); Group 2: moderate activity (1–5 h/week); Group 3: high activity (5–10 h/week); Group 4: very high activity (>10 h/week), in non-HC and HC users. The physical activities/sport disciplines were distributed, as shown in Table 2, into the following categories: strength and speed; team sports and mixed activities; endurance; other types of sport (combat sports and motor skills), with some of the subjects reporting different activities.

**TABLE 1:** Student characteristics.

	Group 1	Group 2	Group 3	Group 4
**N (% of total)**	135 (17.2)	352 (44.7)	197 (25.0)	103 (13.1)
**Non-HC users, n (% per Group)**	56 (41.7)	162 (46.0)	86 (43.7)	48 (46.6)
Age in years (SD)	20.7 (2.2)	20.9 (2.2)	20.6 (2.3)	19.9 (2.0)
Body mass index, kg/m^2^ (SD)	20.7 (3.0)	21.5 (3.2)	21.9 (2.7)	21.8 (2.8)
Age of menarche (SD)	12.5 (1.4)	12.8 (1.4)	12.7 (1.4)	13.1 (1.6)
**Normal menstrual cycle (% of non-HC users)**	43 (76.8)	132 (81.5)	71 (82.6)	29 (60.4)*
*** *** *Without non-hormonal contraceptive (% of NMC)*	34 (79.1)	111 (84.1)	60 (84.5)	25 (86.2)
*** *** *With non-hormonal contraceptive (% of NMC)*	9 (20.9)	21 (15.9)	11 (15.5)	4 (13.8)
**Menstrual disorders (% of non-HC users)**	13 (16.5)	30 (18.5)	15 (17.4)	19 (39.6)*
*** *** *Oligomenorrhea (% of menses)*	5 (38.5)	25 (83.3)	13 (86.7)	9 (47.4)
*** *** *Amenorrhea (% of disorders)*	8 (61.5)	5 (16.7)	2 (13.3)	10 (52.6)
**HC-users, n (% per Group)**	79 (58.5)	190 (54.0)	111 (56.3)	55 (53.3)
*** *** Age in years (SD)	20.8 (2.0)	20.8 (2.1)	20.4 (1.9)	19.5 (1.8)
*** *** Body mass index, kg/m^2^ (SD)	21.8 (5.3)	21.9 (3.4)	21.6 (2.6)	21.0 (1.7)
*** *** Age of menarche (SD)	13.2 (1.7)	12.9 (1.5)	12.8 (1.5)	12.7 (1.3)
**Combined hormonal contraceptive (% of HC users)**	64 (81.0)	140 (73.7)	87 (78.4)	35 (63.6)
*** *** *Oral*	63 (98.4)	138 (98.6)	87 (100.0)	35 (100.0)
*** *** *Vaginal ring*	1 (1.6)	2 (1.4)	0 (0)	0 (0)
**Progestin only contraceptive (% of HC users)**	13 (16.5)	46 (24.2)	23 (20.7)	16 (29.2)
*** *** *Oral*	5 (38.5)	20 (43.5)	11 (47.8)	11 (68.8)
*** *** *IUD*	4 (30.8)	12 (26.1)	7 (30.4)	2 (12.5)
*** *** *Implant*	4 (30.8)	14 (30.4)	5 (21.7)	3 (18.8)
**Unknown hormonal contraceptive (% of HC users)**	2 (2.5)	4 (2.1)	1 (0.9)	4 (7.3)

HC: hormonal contraception; NMC: normal menstrual cycle; IUD: intrauterine device. Group 1: females practicing less than 1 h/week of physical activity; Group 2: females practicing 1–5 h/week of physical activity; Group 3: females practicing 5–10 h/week of physical activity; Group 4: females practicing more than 10 h/week of physical activity.

**TABLE 2:** Prevalence of physical activity and main sports’ discipline reported per Group.

Group (n)	Strength and speed sport (crossfit, fitness, musculation, pilates, powerlifting, weightlifting.)	Endurance sport (athletics, cross-country skiing, cycling, swimming, triathlon.)	Team and mixed sport (badminton, basket, handball, roller, rugby, soccer, table tennis, tennis.)	Combat and others sports (archery, dance, equitation, fencing, gymnastics, judo, shooting, yoga.)	Total
**Group 1¤**	20 (40%) *	16 (32%)	7 (14%)	7 (14%)	50 (100%)
**Group 2**	123 (32%)*	91 (24%)	78 (21%)	87 (23%)	379 (100%)
**Group 3**	44 (21%)	59 (28%)	64 (31%) ^a^	41 (20%)	208 (100%)
**Group 4**	51 (22%)	71 (30%)	72 (31%) ^a^	39 (17%)	233 (100%)
**Total**	238	237	221	174	870

Data are expressed as n (%). 89 females had no physical activity or sport. More than one activity were sometimes mentioned.

*p < 0.05 higher proportion of females practicing strength and speed compared with groups 3 and 4.

^a^ higher proportion of females practicing team sports or mixed activities compared with groups 1 and 2.

We defined a normal menstrual cycle as having an average length of 28 days, with an inter-individual variation of 23–35 days (Hirschberg, 2022), with or without non-hormonal contraceptive, such as a copper intrauterine device.

We defined menstrual cycle disorders as oligomenorrhea (i.e., menstrual cycle length of more than 36 days but less than 3 months) and primary or secondary amenorrhea (i.e., absence of menstrual bleeding for three or more consecutive months (Nattiv et al., 2007)). Dysmenorrhea was not considered in this study as menstrual cycle disorder, but as menses-associated symptoms.

We separated hormonal contraceptives according to the hormones administered: combined hormonal contraceptive (CHC), progestin-only contraceptive or unknown, with the route of administration specified (oral, intrauterine device (IUD), implant or vaginal ring). As there was no menses with continuous HC administration, only oral CHC responses were recorded to analyze menses symptoms during the 7-day washout, and, as explained above, only during the ME phase. Thus, perception of mental and physical symptoms as well as self-reported diet, and analgesic and anti-inflammatory medication intake were investigated over 3 conditions: PMNMC, MENMC and MECHC. NMC and CHC as an index.

### Data analysis

We exported the raw data from Google Forms to Microsoft Excel and performed statistical analyses using Jamovi (version 2.2.5.) and Sigmastat (Software version 3.5). Quantitative data are presented as mean (SD), whereas categorical variables are expressed as a total number and as a percentage. Data were examined for normal distribution before analysis using a Shapiro-Wilk test, and for variance equality using a modified Levene’s test. Based on the results of both tests, since total data had a non-normal distribution, we used non-parametric tests to compare self-perceived symptoms between groups (Groups 1–4), between students (ME_NMC_ vs. ME_CHC_), and between menstrual phases (PM_NMC_ vs. ME_NMC_). The level of statistical significance was set at *p* < 0.05.

We used chi-square analysis with contingency table for qualitative comparisons between groups. If the *p*-value with the four groups included was significant, paired comparisons between groups were done with a chi-square 2 test and *p* was corrected using the Bonferroni method. Thus, the results of these comparisons were significant if *p* < 0.008.

## Results

A total of 834 questionnaires were submitted online. When grouping the majors of female students, 61% were studying sport (*n* = 314, 38%) or health/physical therapy (*n* = 194, 23%). Some questionnaires were excluded from analysis due to incomplete number of hours of physical activities (*n* = 10), type of contraception (*n* = 17) or self-perceived symptoms (*n* = 20). In Part 1, to compare PM_NMC_, ME_NMC_ and ME_CHC_, we used fully completed questionnaires with the following specifications: non-HC users with NMC or oral CHC, physical and mental self-perceived symptoms, self-reported diet and medication. Then, in Part 2, we examined some specific questions that did not require the entire questionnaire on self-perceived symptoms to be completed, e.g., reasons for HC use, knowledge of oral contraception taken, school and sport absenteeism or adaptation, perceived relationship between self-perceived symptoms and physical activity during PM and ME phases, and the use of feminine hygiene products. Groups 1 and 2 included a higher proportion of females practicing strength and speed (40% in Group 1, 32% in Group 2, 21% in Group 3% and 22% in Group 4, *p* < 0.001), whereas more females in Groups 3 and 4 engaged in team or mixed sports (14% in Group 1, 21% in Group 2, and 31% in Groups 3 and 4, *p* = 0.002). There was no difference between groups in the number of female students practicing essentially endurance sports (24%–32%, *p* = 0.27) or combat and other types of sport (archery, dance, equitation, fencing, gymnastics, judo, shooting, yoga) (14%–23%, *p* = 0.19).

## Part 1

### Sample size and participant characteristics for complete questionnaires

We included a total of 787 surveys in the main analysis (Group 1 *n* = 135, Group 2 *n* = 352, Group 3 *n* = 197 and Group 4 *n* = 103). Detailed information on the students’ characteristics is presented in Table 1. There was no significant difference between the groups in terms of body mass index, age of menarche, with or without HC. Group 4 showed a higher proportion of females having menstrual cycle disorders compared with the other three groups (Group 1: 16.5%, Group 2: 18.5%, Group 3: 17.4% and Group 4: 39.6%, *p* < 0.05). The number of females using HC varied from 53% to 59% and was not different between groups (*p* = 0.13). Most of the females in the four groups used CHC (64%–81%), primarily via oral intake (99%–100%).

We excluded females with menstrual disorders and HC- non CHC users from the next analysis. Detailed information on the nature of perceived physical and mental symptoms in the remaining females (non-HC users with normal menstrual cycle and CHC users) are reported in Table 3.

**TABLE 3:** Self-perceived physical and mental symptoms (%) reported.

A. In NMC students during PM phase					
Symptoms	Group 1	Group 2	Group 3	Group 4	*p*-value
n (PM_NMC_)	43	132	71	29	-
**Physical (%)**					
Appetit loss	7.0	5.3	5.6	0	**0.02** ^α^
Bad skin	34.9	40.9	36.6	10.3	0.45^α^
Bloating	20.9	19.7	16.9	6.9	0.48^α^
Breast tendemess	27.9	40.2	29.6	13.8	0.34^α^
Constipation	9.3	7.6	5.6	13.8	0.52
Cramps	7.0	11.4	7.0	3.4	**0.02** ^α^
Decrease performance	7.0	8.3	7.0	3.4	0.11^α^
Dizziness	4.7	4.5	7.0	3.4	0.11^α^
Fatigue	32.6	32.6	31.0	17.2	0.21^α^
Gastrointestinal disturbances	11.6	12.9	5.6	3.4	**0.01** ^α^
Headache and migraine	20.9	27.3	16.9	24.1	**0.01** ^α^
Lower back pain	9.3	23.5	19.7	10.3	0.41^α^
Lower belly pain	23.3	36.4	39.4	31.0	**0.01** ^α^
Sick/Nauseous	2.3	6.1	8.5	0	0.11^α^
Weight gain	7.0	9.1	7.0	3.4	0.39^α^
Weight loss	0	0	1.4	0	0.46
**Mental (%)**					
Anxiety attack	4.7	9.1	0	3.4	0.12
Irritability	23.3	25.8	25.4	13.8	0.76^α^
Sleep disturbance	18.6	12.1	8.5	6.9	**0.02** ^α^
Stress	14.0	16.7	9.9	13.8	0.15^α^
Tearfulness	9.3	21.2	9.9	10.3	0.71^α^

B. In NMC students during ME phase					
Symptoms	Group 1	Group 2	Group 3	Group 4	*p*-value
N (ME_NMC_)	43	132	71	29	-
**Physical (%)**					
Appetit loss	14.0	12.1	7.0	0	0.15^δβ^
Bad skin	46.5	49.2	46.5	37.9	0.74^δ^
Bloating	30.2	31.1	31.0	20.7	0.73^δβ^
Breast tendemess	32.6	40.9	38.0	20.7	0.21^δβ^
Constipation	11.6	10.6	9.9	6.9	0.92^β^
Cramps	37.2	34.8	23.9	13.8	0.06^δβ^
Decrease performance	30.2	19.7	12.7	20.7	0.15^δβ^
Dizziness	11.6	17.4	7.0	6.9	0.13^δβ^
Fatigue	58.1	46.2	46.5	41.4	0.47^δβ^
Gastrointestinal disturbing	41.9	46.2	32.4	17.2	**0.02** ^δβ^
Headache and migraine	39.5	30.3	19.7	13.8	**0.04** ^δβ^
Lower back pain	48.8	43.9	43.7	37.9	0.83^δβ^
Lower belly pain	76.7	71.2	66.2	48.3	0.06^δβ^
Sick/Nauseous	11.6	15.2	11.3	0	0.16^δβ^
Weight gain	9.3	12.1	15.5	13.8	0.80^δβ^
Weight loss	0	3.0	0	0	0.22^δβ^
**Mental (%)**					
Anxiety attack	9.3	8.3	0	6.9	0.09
Irritability	30.2	31.8	26.8	31.0	0.90^δβ^
Sleep disturbance	16.3	12.1	5.6	3.4	0.15^δ^
Stress	30.2	16.7	15.5	17.2	0.20^δ^
Tearfulness	27.9	19.7	16.9	27.6	0.42^δβ^

C. In CHC students during ME phase					
Symptoms	Group 1	Group 2	Group 3	Group 4	*p*-value
N (ME_CHC_)	92	220	126	74	-
**Physical (%)**					
Appetit loss	7.9	3.6	1.6	0	0.06^α^
Bad skin	22.2	23.6	5.5	18.8	0.55^α^
Bloating	12.7	15.7	14.3	12.5	0.96^α^
Breast tendemess	17.5	18.1	16.7	15.6	0.98^α^
Constipation	3.2	4.7	3.8	9.4	0.67^α^
Cramps	19.0	7.1	11.9	15.6	0.06^α^
Decrease performance	11.1	8.7	9.5	3.1	0.55^α^
Dizziness	3.2	2.4	6.0	3.1	0.56^α^
Fatigue	31.7	22.8	17.9	25.0	0.19^α^
Gastrointestinal disturbing	22.2	19.7	14.3	9.4	0.28^α^
Headache and migraine	28.6	23.6	14.3	6.3	**0.02** ^α^
Lower back pain	27.0	21.3	23.8	15.6	0.46^α^
Lower belly pain	38.1	35.4	32.1	31.3	0.74^α^
Sick/Nauseous	6.3	7.1	3.6	6.3	0.78^α^
Weight gain	6.3	7.1	4.8	6.3	0.93^α^
Weight loss	0	0.8	0	0	0.72
**Mental (%)**					
Anxiety attack	4.8	7.1	6.0	6.3	0.97
Irritability	23.8	20.5	21.4	18.8	0.83^α^
Sleep disturbance	6.3	9.4	8.3	9.4	0.95
Stress	19.0	15.7	13.1	9.4	0.50
Tearfulness	20.6	14.2	14.3	9.4	0.36^α^

PM: premenstrual phase; ME: menstruation phase; NMC: normal menstrual cycle; CHC: combined hormonal contraceptive; Group 1: females practicing less than 1 h/week of physical activity; Group 2: females practicing 1–5 h/week of physical activity; Group 3: females practicing 5–10 h/week of physical activity; Group 4: females practicing more than 10 h/week of physical activity.

*p*-value: Group effect *p*-value.

Menstrual phase *p*-value. ^δ^p<0.05 with PM_NMC_; ^α^p<0.05 with ME_NMC_.

Contraception or not *p*-value. ^α^p<0.05 with ME_NMC_; ^β^p<0.05 with ME_CHC_.

### Effect of physical activity level on self-perceived symptoms, self-reported diet, supplement and medication use

**FIGURE 1:**
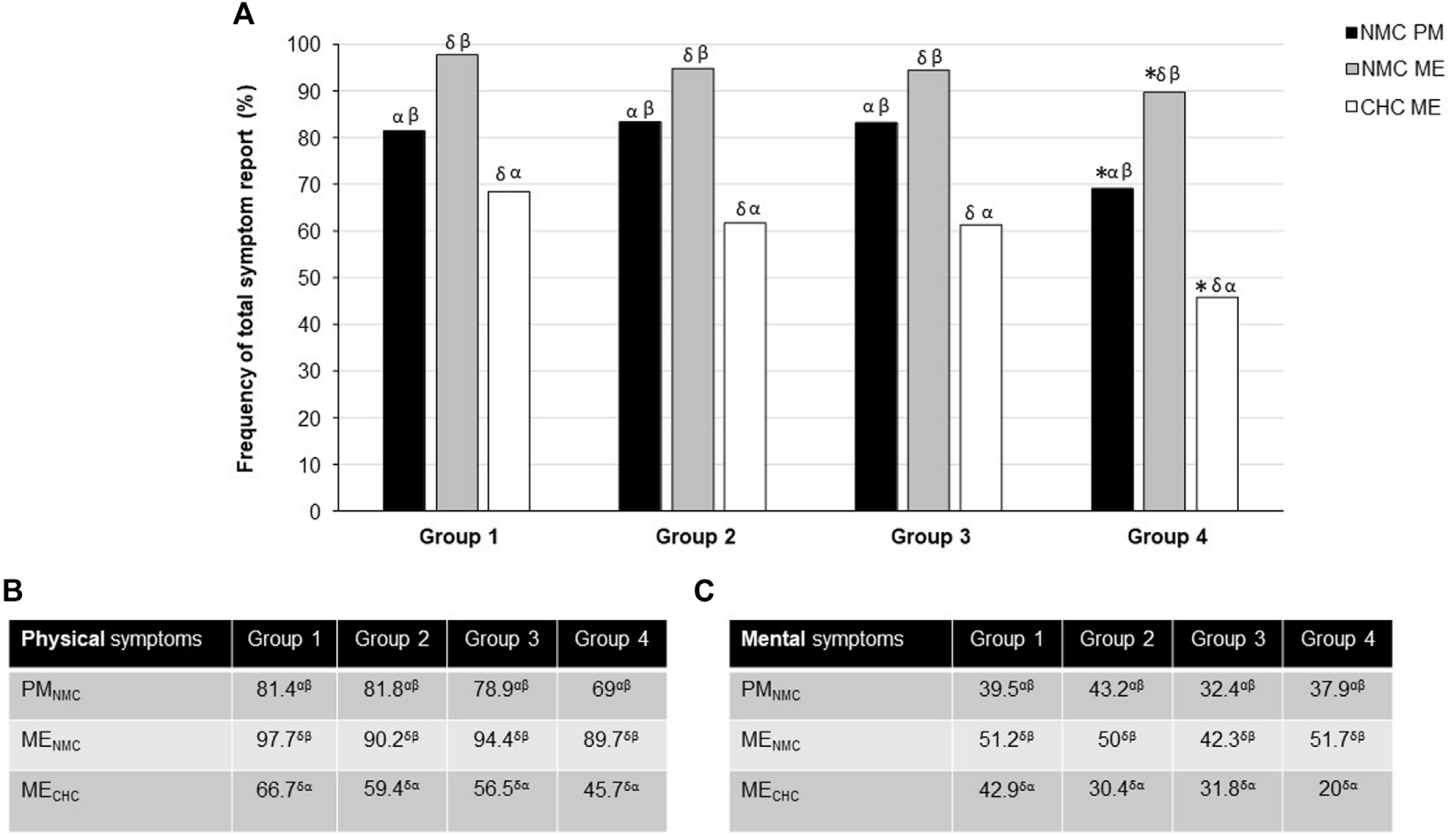
Frequency of total **(A)**, physical **(B)** and mental **(C)** self-perceived symptoms according to the groups, menstrual phase and use of contraception. CHC: Combined hormonal contraceptive; NMC: Normal menstrual cycle; PM: premenstrual phase; ME: menstrual phase. *p < 0.05 with Groups 1 and 2 at a same menstrual phase and contraception use; ^δ^p<0.05 with PM_NMC_; ^α^p<0.05 with ME_NMC_; ^β^p<0.05 with ME_CHC._ Data are expressed as percentage of female students reporting at least one symptom in each category.

**FIGURE 2:**
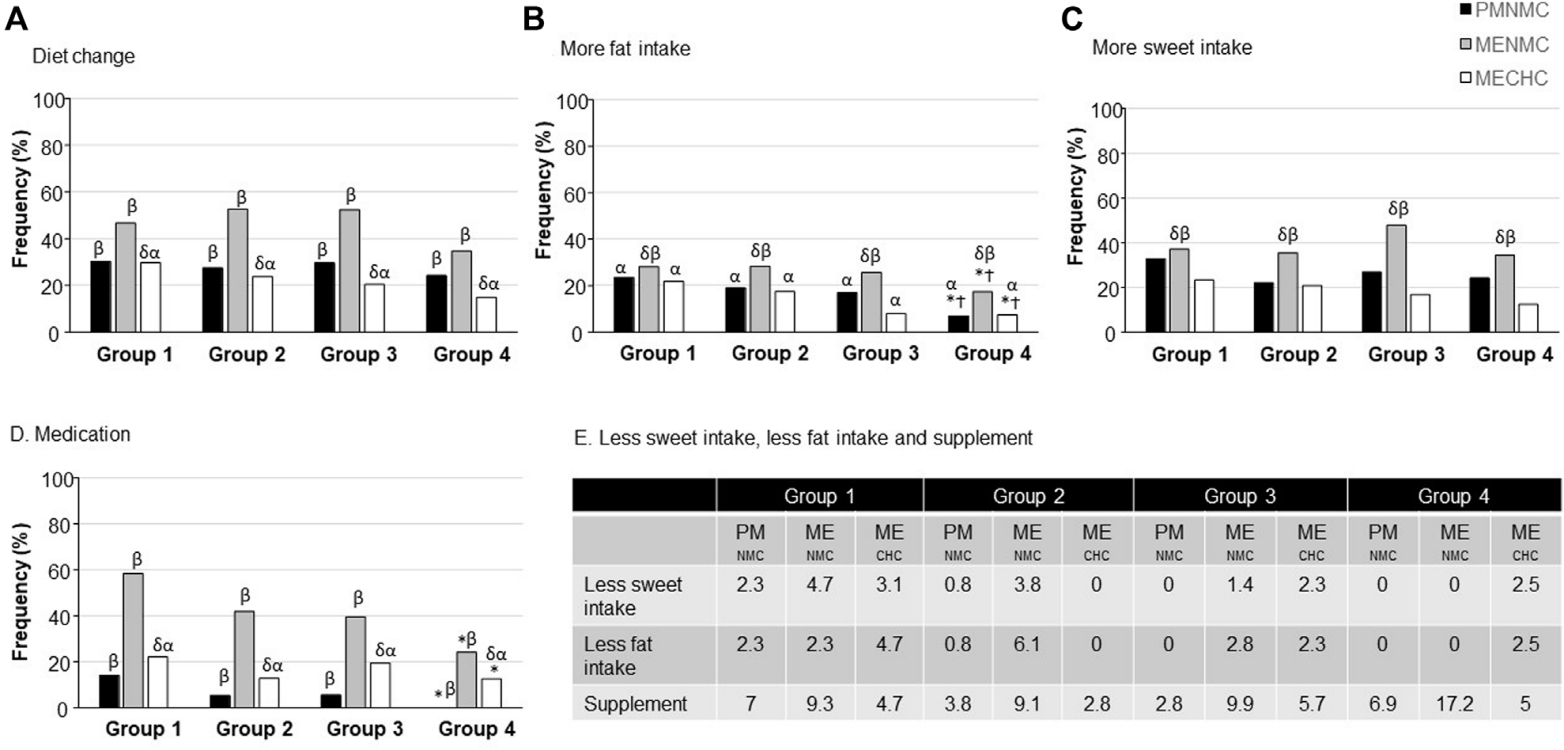
Self-reported dietary changes, supplement and medication consumption during ME phase **(A)** in NMC students and **(B)** in CHC students. CHC: Combined hormonal contraceptive; NMC: Normal menstrual cycle; ME: menstrual phase. *p < 0.05 with Group 1; Ϯ *p* < 0.05 with Group 2 at a same menstrual phase and contraception use; ^δ^p<0.05 with PM_NMC_; ^α^p<0.05 with ME_NMC_; ^β^p<0.05 with ME_CHC._ Data are expressed as percentage of female students reporting symptom in each category.

There were significantly fewer total self-perceived symptoms in Group 4 compared with Groups 1 and 2 (*p* = 0.016) whatever the condition (PM_NMC_, ME_NMC_ and ME_CHC_) (Figure 1). The detail for each mental and physical symptom is presented in Table 3. Self-reported fat intake was less altered in Group 4 (*p* < 0.05) during PM and ME phases compared with Groups 1 and 2 (Figure 2). Finally, Group 1 used significantly more analgesic/anti-inflammatory medication than Group 4 (*p* = 0.048) (Figure 2).

### Effects of contraception and menstrual cycle phase on self-perceived symptoms, self-reported diet, supplement and medication use

Fewer self-perceived symptoms were reported in PM_NMC_ compared with ME_NMC_ (*p* < 0.05) with no significant group effect. The detail for each mental and physical symptom is presented in Table 4. Fewer self-reported diet alterations, total, physical, and mental symptoms were also found in ME_CHC_ vs. both PM_NMC_ and ME_NMC_ (*p* < 0.05; Figures 1, 2) regardless of physical activity. There was a more marked self-reported diet, sweet and fat intake alteration in ME_NMC_ than in PM_NMC_ and ME_CHC_ (*p* < 0.05). There was greater medication use in ME_NMC_ vs. ME_CHC_ (*p* < 0.05) (Figure 2) and in ME_CHC_ vs. PM_NMC_ (*p* < 0.05).

## Part 2

**FIGURE 3:**
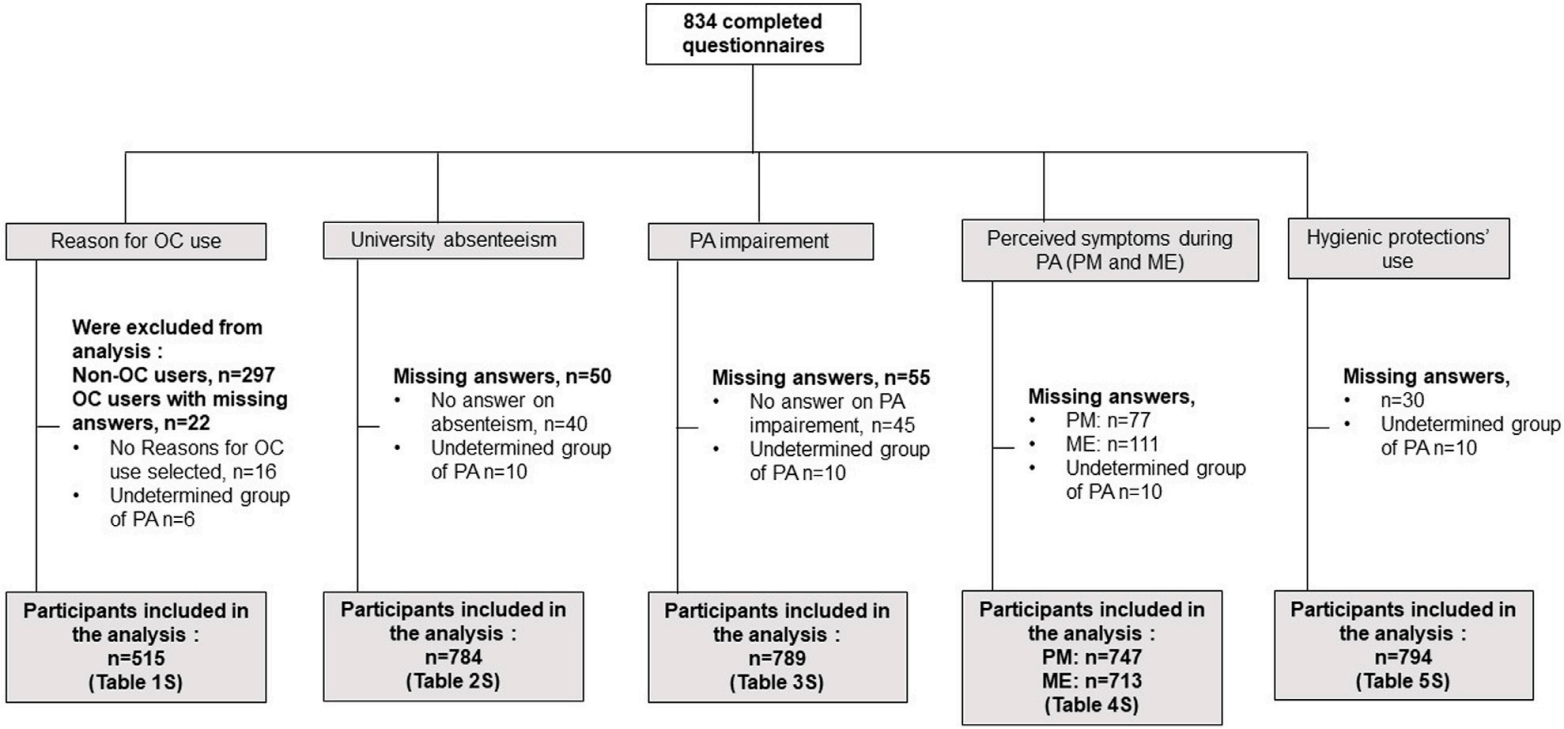
Self-reported dietary changes **(A)**, more fat intake **(B)**, more sweet intake **(C)**, medication consumption **(D)**, less sweet and fat intake and supplement **(E)**. CHC, Combined hormonal contraception; NMC, Normal menstrual cycle; ME, menstrual phase; PM, premenstrual phase.

The flow chart for part 2 of the study is presented on Figure 3.

### Reasons for taking HC

Of 537 females taking HC, 6 did not report their level of physical activity and 16 did not state why they take HC. Of the remaining 515 HC users, no between-group difference was observed concerning the reasons for taking contraception (Supplementary Table S1). Contraception was the main reason (79% in Group 1, 75% in Group 2, 78% in Group 3% and 83% in Group 4, *p* = 0.36) followed by therapeutic need (51% in Group 1, 60% in Group 2, 54% in Group 3% and 63% in Group 4, *p* = 0.24) (Supplementary Table S1). Therapeutic reasons were: to have regular menses (35%–43%), reduce premenstrual symptoms (20%–25%), stop menses due to a pathology or for convenience (10%–16%), and reduce acne (8%–14%). Of the 386 females who took the pill as HC, 202 (55%) did not know what type of pill it was (CHC or progestin). Of these, 188 named the brand, and 14 did not know which brand they took.

### School/university absenteeism

Seven hundred eighty-four females completed the questionnaire for the amount of physical activity, contraception use and school absenteeism (Supplementary Table S2). In non-HC only, the proportion of school absenteeism during PM and/or ME phase was significantly lessened in group 3 compared with group 1 (*p* = 0.002). It was of 41% in Group 1, 27% in Group 2, 13% in Group 3, and 17% in Group 4 (*p* = 0.005). In females using HC, absenteeism in the four groups varied from 13% to 23% and was not significantly different between groups (*p* = 0.17).

### Impact of the menstrual cycle on physical activity

Of the 789 participants who answered the questions on the amount of physical activity, contraception or not and continuation, suspension or adaptation of training, 376 (48%) reported they had to stop or adapt their training/physical activity (activity impairment) during or just before menses (Supplementary Table S3). Fifty percent of the females in Group 1, 58% in Group 2, 37% in Group 3 and 34% in Group 4 (*p* < 0.001) reported having to adapt their physical activity during ME. The percentage was higher in Group 2 compared with Groups 3 (*p* < 0.001) and 4 (*p* < 0.005), with or without HC. The main adaptations reported were suspending all or some physical activities or decreasing the intensity for the required time. The main activities that are stopped during menses are cardio activities and ones working abdominal muscles (fitness and weight training), swimming and water sports with the main reason being not wanting to wear tampons, and running, because of discomfort and weakness. The main reasons given are various degrees of pain and symptoms, fatigue and lack of energy, strength and power, heavy bleeding, fear of bloodstains on clothing, but also a lack of desire/motivation to engage in sports during menses and/or the week before.

### Increased or decreased perception of symptoms associated with physical activity during PM and ME

During PM, the proportion of students reporting a decrease (14%–22%) or increase (8%–12%) in self-perceived symptoms due to physical activity was not significantly different between groups (*p* = 0.42 and *p* = 0.71, respectively). During ME, in non-HC only, Groups 3 and 4 had a higher proportion of females reporting a decrease in menstrual symptoms with physical activity compared with Group 2 (25% for Group 2, 50% for Group 3% and 38% for Group 4, *p* < 0.001 for both) (Supplementary Table S4).

### Feminine hygiene product use

Eight hundred females completed the questions on the amount of physical activity and use of feminine hygiene products. Six females used no protection as they had no menses. Of the 794 remaining females, all four groups used primarily menstrual pads (72%–76%), with no difference between groups (*p* = 0.84) (Supplementary Table S5). Menstrual cups were used in the same proportion (6%–11%) in all four groups (*p* = 0.43). Tampons were used significantly more by Group 4 compared with Groups 1 and 2 (32% in Group 1, 33% in Group 2, 41% in Group 3% and 52% in Group 4, *p* = 0.003 and *p* < 0.001, respectively). Finally, menstrual underwear was less used by Group 4 than Group 2 (26% in Group 1, 30% in Group 2, 21% in Group 3 and 16% in Group 4) (*p* = 0.005).

## Discussion

Our main results are: 1) the perception of menstrual cycle symptoms are lower with CHC use in female students whatever their physical activity; 2) prevalence of physical and mental self-perceived symptoms in NMC and in CHC female students were less in the group reporting 10 h/week of physical activity. Females who trained five or more hours/week also reported a lesser impact of menses on their physical and academic activities than did female students who trained less, especially females not using HC.

In this study performed in France, most of the female students engaged in physical activity and sports between 1 and 10 h/week (70%), with relatively few inactive students (17%) or students practicing more than 10 h/week (13%). There was no significant difference in age, age at menarche or body mass index, regardless of the level of practice. It can be noticed the relatively low BMI that may be partly explained by the high proportion of females studying sports and health/physical therapy but these data are quite similar to the literature, which reports that female students aim to be physically active primarily to maintain fitness, enjoy good health, have fun, relax and meet friends (Carballo-Fazanes et al., 2020). Also, the increase in oligomenorrhea or amenorrhea in NMC students engaged in physical activity for more than 10 h/week (Group 4) observed in our study has been widely described in the literature (Castanier et al., 2021; Gimunová et al., 2022), with multifactorial origins. The use of oral or non-hormonal contraceptive among the students in our study does not vary according to their physical activity level, with the percentage of use varying between 53% and 59%. In agreement with the literature (Birgisson et al., 2015; Balakrishnan et al., 2023), the majority of females used CHC, i.e. 64%–81% of HC, mainly by oral route (98%–100%). The use of progestin-only contraceptives represents only 17%–29% of HC use, with various routes of administration: oral, IUD or implant. Few studies report female students’ reasons for taking HC. A recent study reported that 68% of oral contraception users took it for a contraceptive purpose and 32% for a therapeutic purpose (Nielson and Beltz, 2021). We addressed the question less dichotomously in our study, and the responses show that many students use contraception for both reasons. More than 75% of respondents choose HC for contraception and more than 50% for therapeutic reasons. When therapeutic reasons were mentioned, they were to regulate the menstrual cycle (35%–48%), reduce premenstrual symptoms (20%–25%), stop menses (for convenience or due to a pathology: 10%–16%) and fight acne (8%–14%). It has been previously reported that 69% of eastern European female university students believed they needed monthly menses to be healthy (Szűcs et al., 2017), potentially explaining the main reason mentioned above. However, about 40% do not wish to have monthly bleeding (Nappi et al., 2016; Fiala et al., 2017; Szűcs et al., 2017), and would prefer longer intervals between menses due the self-perceived symptoms of menses and their effects on daily life. Consequently, a continuous or extended cycle through contraceptive manipulation may be chosen (Edelman et al., 2014), but until now few studies have reported the extent of this practice. Last, the effects of oral contraceptive for acne have been recently reviewed and despite a lack of consensus, some forms of acne could benefit from it (Mavranezouli et al., 2022).

At least 70% of NMC students, whatever their physical activity level, reported self-perceived symptoms at the end of the luteal phase and even more markedly during menses (between 90% and 98%). During the PM phase, the most frequently described self-perceived physical symptoms are fatigue, headache and migraine, breast tenderness and abdominal pain, whereas dysmenorrhea predominates during the ME phase, with lower back and gastro-intestinal disturbance as well. The most common self-perceived mental symptoms, less frequently reported by NMC students in both the PM and ME phases, are irritability, stress and tearfulness. These data support the literature, although there are significant variations in the prevalence of self-reported symptoms of the menstrual cycle, probably related to the populations investigated (Czajkowska et al., 2015; Czajkowska et al., 2019; Castanier et al., 2021). Large studies conducted with female students from various European countries showed that despite not suppressing menstrual symptoms, CHC was associated with reduced number, severity and frequency (Lete et al., 2017) in accordance with previous published data (Sadler et al., 2010; Ekenros et al., 2019; Robakis et al., 2019). We made a similar observation, and the perception of all self-perceived symptoms and alterations in self-reported dietary intake were lower during the ME in CHC users, compared with NMC, regardless of the amount of physical activity. The hypothesis is lesser hormonal variations during this exogenous intake of synthetic estrogens and progestins inhibiting ovulation, compared with females without contraception. However, it is interesting to note that the level of physical activity appears to modulate these CHC effects. Indeed, first, lower alteration of self-reported fat intake was observed in the two groups with greater physical activity. Moreover, there was significantly less self-perceived mental symptoms during the ME phase only in the CHC group with very high physical activity. To our knowledge, this last point has never been highlighted previously and new research with biological analyses appears necessary to understand the repercussions of the association of high physical activity and CHC on the neurotransmitters involved in stress, anxiety and self-reported fat intake.

Finally, our data showed that in the group reporting more than 10 h/week of physical activity, there were both fewer self-perceived symptoms and self-reported dietary changes during the PM and ME phases in females with CHC and in those not using HC with normal menstrual cycles. Increased use of analgesic and anti-inflammatory drugs was also observed during both the PM and ME phases in the inactive group (<1 h/week) compared with Group 4. The literature is not unanimous on the benefits of intense physical activity on menstrual and premenstrual self-perceived symptoms. Some studies conducted with adolescent girls reported no change in the frequency or severity of self-perceived symptoms in athletes and their inactive peers (Takeda et al., 2016; Czajkowska et al., 2019), while others found an increase during ME (Czajkowska et al., 2015). A systematic review analyzing randomized control trials suggested that 45 min to 1 h of activity three times per week may be effective in reducing dysmenorrhea (Armour et al., 2019a). Our results suggest that the female students in Group 3 practicing regular physical activity at least 5 h/week reported a lesser impact of ME self-perceived symptoms on their physical and university activities, especially those without HC. As the proportion of self-perceived symptoms did not significantly change with those training less than 5 h/week, we can hypothesize that the severity of self-perceived symptoms may be lessened in active females or that physical activity helps them to bear their symptoms, as declared by 37% compared with 23% in Groups 1 and 2. Huang *et al.* (Huang et al., 2022) showed a link between hormone level and imbalance, and various symptoms in dysmenorrheic females. Concomitant to the amelioration of symptoms and pain severity with physical activity, the authors showed a decrease in estradiol and prolactin, and an increase in progesterone and cortisol after a training program, reaching values similar to those of non-dysmenorrheic females (Huang et al., 2022). They also noted a decrease in prostaglandin metabolites and high sensitivity C-reactive protein, suggesting a hormonal and inflammatory modulation effect of physical activity. Their program was composed of high-intensity training, and to our knowledge there is no data on a comparison of inflammatory parameters related to the menstrual cycle between various combinations of duration, intensity and frequency of physical activity in young females.

In our study, school absenteeism ranged from 13% to 41%. The much lower prevalence of school absenteeism in our study compared with 60.5% of Spanish nursing students who reported skipping classes during menses (Fernández-Martínez et al., 2019b) is probably due to the different characteristics of the two populations (nurses only vs*.* students from various disciplines, non-HC users were the majority in their study and only 12% used CHC). The authors reported that dysmenorrhea increases the risk of absenteeism by 3–6 times depending on the type of symptom and the use of CHC to decrease it, but only 12% of the students included in their study used CHC (Fernández-Martínez et al., 2019b). In our study, HC use, including CHC but also other contraceptive means, did not reduce absenteeism in any group. In addition, school absenteeism during PM and ME was lower in non-HC females who train more than 5 h/week compared with those who train for less time. In addition to school absenteeism, the ability to train normally was impaired in 48% of female students who had to stop or decrease the intensity of PA during ME or PM, especially in the group training less (1–5 h/week). In a similar study led in Ethiopia, the authors reported 38% of female students had a limited sport participation during PM or ME (Hailemeskel et al., 2016). A recent study led in Greece showed female student with severe menses pain reported more frequently postponing physical activities (51%), compared with 33.7% and 23.2% in those with moderate and mild pain, respectively (Vlachou et al., 2019). In our study as in the previous one, despite not assessing self-perceived symptoms intensity, pain, and then lack of energy and fatigue were the most cited explanation for limitation of PA, with some activities most impacted, like running, jumping or high intensities activities (Vlachou et al., 2019; Fernández-Martínez et al., 2020). Additional limited activities and reasons have been cited in our study and fitness/weight training as it works the abdominals, water sports/swimming for those who do not want to wear tampons, fear of bloodstains on clothing but also a lack of desire/motivation to engage in sports during menses and/or the week before have been mentioned by few students. In addition to be associated with academic performance (not studied here), menses may also be linked to the inability to normally practice a physical activity. 38% of the students included in our study are in sports science, and sports practice is crucial in their university curriculum, and represents an important percentage of the grade in the first years. Thus, especially for the latter, it is urgent to take measures so that the menstrual cycle does not impact schooling and academic results. Finally, it is interesting to note that the level of physical activity is associated the type of feminine hygiene product used, with female students who train more than 10 h/week using more tampons than those who work out less than 5 h/week. Further studies are needed to investigate knowledge, advantages and disadvantages considered by the students when choosing feminine hygiene products, since some feared getting blood on their clothes and others suspended their water sport activities during their period because they did not want to use tampons.

Our study’s methodology has inherent limitations classical linked to a self-made questionnaire use. The hours of training per week, the self-reported diet intake and the self-perceived symptoms were reported by students but were not measured or verified. The intensity of training or duration of each session is missing and would have provided some additional data, although intensity is probably linked to the reported number of training hours per week. The severity of self-perceived symptoms was not assessed, and it is possible that despite no change in the number of symptoms with physical activity in Group 3, severity may have decreased, causing them to have less of an impact on social activities. Last, this type of study (i.e., online questionnaire) is unable to prove any causal effect. However, our methodology was quite similar to previous studies on the topic that are cited in this manuscript.

## Conclusion and perspectives

In conclusion, the self-reported perception of menstrual cycle symptoms during both the premenstrual and menstrual phases in students without HC was lower with very high physical activity. The self-perceived symptoms were also lower with CHC use at all physical activity levels, but more markedly in high physical activity female students. Moreover, those training more than 5 h/week also reported a beneficial effect on attendance at university and involvement in physical activities. Finally, our study cannot establish a causality link and it remains to prove that the level of physical activity and the use of hormonal contraception led female students to have fewer self-perceived symptoms.

## Data Availability

The raw data supporting the conclusions of this article will be made available by the authors, without undue reservation.
