# Supplementary material for: Physical activity and combined hormonal contraception: association with female students’ perception of menstrual symptoms
Source: Front Physiol. 2023 May 17;14:1185343. doi: 10.3389/fphys.2023.1185343 (PMC10230043; doi:10.3389/fphys.2023.1185343)
Supplement: Supplementary file 1 [file Table1.DOCX]

**Table 1S. Reasons for contraceptive use**

| **Reason for HC** | **Group 1** | **Group 2** | **Group 3** | **Group 4** | **p-value** |
| --- | --- | --- | --- | --- | --- |
| n HC-users | 90 | 230 | 131 | 64 |  |
| **Contraception n (%)** | 71 (79) | 172 (75) | 102 (78) | 53 (83) | 0.36 |
| **Therapeutic use n (%)** | 46 (51) | 137 (60) | 71 (54) | 40 (63) | 0.24 |
| *To have regular menses* | 39 (43) | 87 (38) | 46 (35) | 30 (47) | 0.57 |
| *Stop menstruations (either due to a pathology or not)* | 11 (12) | 24 (10) | 14 (11) | 10 (16) | 0.44 |
| *Acne* | 13 (14) | 26 (11) | 11 (8) | 9 (14) | 0.82 |
| *Pre-menstrual symptoms* | 18 (20) | 53 (23) | 28 (21) | 16 (25) | 0.60 |

HC: hormonal contraception

**Table 2S. Scholar absenteeism**

|  | **Group 1** | **Group 2** | **Group 3** | **Group 4** | **p-value** |
| --- | --- | --- | --- | --- | --- |
| n | 130 | 345 | 204 | 105 |  |
| Scholar absenteeism n (%) | 38 (29) | 80 (23) | 28 (14) | 15 (14) | 0.003 |
| **Non-HC** | 44 | 124 | 70 | 41 |  |
| Scholar absenteeism n (%) | 18 (41%) | 33 (27%) | 9 (13%)* | 7 (17%) | 0.005 |
| **HC** | 86 | 221 | 134 | 64 |  |
| Scholar absenteeism n (%) | 20 (23%) | 47 (21%) | 20 (15%) | 8 (13%) | 0.17 |

*p<0.008 with group 1; HC: hormonal contraception

**Table 3S. Physical activity impairement**

|  | **Group 1** | **Group 2** | **Group 3** | **Group 4** | **p-value** |
| --- | --- | --- | --- | --- | --- |
| n | 125 | 351 | 205 | 108 |  |
| Physical activity impairement n (%) | 62 (50%) | 202 (58%) | 75 (37%)δ | 37 (34%)δ | <0.001 |
| **Non-HC** | 40 | 126 | 71 | 44 |  |
| Physical activity impairement n (%) | 19 (48%) | 73 (58%) | 26 (37%) δ | 15 (23%) | 0.007 |
| **HC** | 85 | 225 | 134 | 64 |  |
| Physical activity impairement n (%) | 43 (51%) | 129 (57%) | 49 (37%) δ | 22 (34%) δ | <0.001 |

δ p<0.008 with group 2 ; HC: hormonal contraception

**Table 4S. Perceived symptoms with physical activity (A) during PM, (B) during ME.**

| **(A)** | **Group 1** | **Group 2** | **Group 3** | **Group 4** | **p-value** |
| --- | --- | --- | --- | --- | --- |
| n | 111 | 332 | 202 | 102 |  |
| Increase **PMS** with physical activity n (%) | 10 (9) | 34 (10) | 16 (8) | 12 (12) | 0.71 |
| Decrease **PMS** with physical activity n (%) | 15 (14) | 55 (17) | 38 (19) | 22 (22) | 0.42 |

| **(B)** | **Group 1** | **Group 2** | **Group 3** | **Group 4** | **p-value** |
| --- | --- | --- | --- | --- | --- |
|  |  |  |  |  |  |
| n | 106 | 315 | 197 | 95 |  |
| Increase **menstrual** symptoms with physical activity n (%) | 15 (14) | 76 (24) | 35 (18) | 13 (14) | 0.034 |
| Decrease **menstrual** symptoms with physcial activity n (%) | 24 (23) | 71 (23) | 72 (37) δ | 36 (38) δ | <0.001 |
| ***Non-HC*** | 32 | 112 | 70 | 40 |  |
| *Decrease menstrual symptoms with physical activity n (%)* | 8 (25%) | 28 (25%) | 35 (50%) δ | 15 (38%) δ | <0.001 |
| ***HC*** | 74 | 203 | 127 | 55 |  |
| *Decrease menstrual symptoms with physical activity n (%)* | 16 (22%) | 43 (21%) | 37 (29%) | 21 (38%) | 0.044 |

δ p<0.008 with group 2 ; PMS: premenstrual symptoms; HC: hormonal contraception

**Table 5S. Hygienic protections’ use**

|  | **Group 1** | **Group 2** | **Group 3** | **Group 4** | **p-value** |
| --- | --- | --- | --- | --- | --- |
| n | 134 | 348 | 206 | 106 |  |
| Sanitary pads n (%) | 102 (76) | 262 (75) | 149 (72) | 80 (75) | 0.84 |
| Sanitary tampons n (%) | 43 (32) | 116 (33) | 84 (41) | 55 (52) * δ | 0.002 |
| Cups n (%) | 15 (11) | 38 (11) | 20 (10) | 6 (6) | 0.43 |
| Menstrual panties n (%) | 35 (26) | 106 (30) | 44 (21) | 17 (16) δ | 0.010 |

*p<0.008 with group 1; δ p<0.008 with group 2
